# The central and biodynamic role of gut microbiota in critically ill patients

**DOI:** 10.1186/s13054-022-04127-5

**Published:** 2022-08-18

**Authors:** Hannah Wozniak, Tal Sarah Beckmann, Lorin Fröhlich, Tania Soccorsi, Christophe Le Terrier, Aude de Watteville, Jacques Schrenzel, Claudia-Paula Heidegger

**Affiliations:** 1 Division of Intensive Care, Department of Acute Medicine, Geneva University Hospitals and University of Geneva, Geneva, Switzerland; 2 Division of Anesthesiology, Department of Acute Medicine, Geneva University Hospitals and University of Geneva, Geneva, Switzerland; 3 Emerging Antibiotic Resistance Unit, Medical and Molecular Microbiology, Department of Medicine, University of Fribourg, Fribourg, Switzerland; 4 Genomic Research Laboratory, Service of Infectious Diseases, Geneva University Hospitals and University of Geneva, Geneva, Switzerland

**Keywords:** Gut microbiota, Dysbiosis, Gut–organ axis, Critical care

## Abstract

Gut microbiota plays an essential role in health and disease. It is constantly evolving and in permanent communication with its host. The gut microbiota is increasingly seen as an organ, and its failure, reflected by dysbiosis, is seen as an organ failure associated with poor outcomes. Critically ill patients may have an altered gut microbiota, namely dysbiosis, with a severe reduction in “health-promoting” commensal intestinal bacteria (such as Firmicutes or Bacteroidetes) and an increase in potentially pathogenic bacteria (e.g. Proteobacteria). Many factors that occur in critically ill patients favour dysbiosis, such as medications or changes in nutrition patterns. Dysbiosis leads to several important effects, including changes in gut integrity and in the production of metabolites such as short-chain fatty acids and trimethylamine N-oxide. There is increasing evidence that gut microbiota and its alteration interact with other organs, highlighting the concept of the gut–organ axis. Thus, dysbiosis will affect other organs and could have an impact on the progression of critical diseases. Current knowledge is only a small part of what remains to be discovered. The precise role and contribution of the gut microbiota and its interactions with various organs is an intense and challenging research area that offers exciting opportunities for disease prevention, management and therapy, particularly in critical care where multi-organ failure is often the focus. This narrative review provides an overview of the normal composition of the gut microbiota, its functions, the mechanisms leading to dysbiosis, its consequences in an intensive care setting, and highlights the concept of the gut–organ axis.

## Background: why focus on the gut microbiota in intensive care patients?

The digestive tract contains a considerable number of microorganisms that are in constant communication and symbiosis with their host. They play a major role both in health and in the pathogenesis of many diseases such as inflammatory, cardiovascular or metabolic diseases when dysbiosis occurs [1–3], i.e. when the composition of the gut microbiota is altered.

Critically ill patients are often instable with multi-organ damage. They undergo a major state of stress mediated by endocrine, immunological, neuronal and inflammatory mechanisms [4]. In addition, the gut microbiota is under tremendous pressure due to various factors such as medications, critical illness or the discontinuation of the normal diet [5]. More recently, the gut microbiota is more considered as a dynamic organ and its failure, reflected by dysbiosis, as an organ failure, associated with poor outcomes [5–8]. It is therefore urgent to understand the mechanisms of its evolution and its involvement in critical illnesses.

Actual evidence on the gut microbiota comes either from animal models or from human studies. Murine models have different gut physiology from that of large mammalian models. This must be taken into account when extrapolating results from murine models to humans [9–11]. However, these data allow us to better understand the gut microbiota and its dynamic changes.

Our aim is to provide an overview of the normal composition of the gut microbiota, its functions, the concept of the gut–organ axis, the mechanisms leading to dysbiosis and its consequences in the intensive care units (ICU).

## Normal composition of the gut microbiota and its evolution in intensive care

### Normal composition of the gut microbiota

While there is currently no definition of a “normal” microbiota [12, 13], many factors, such as diet, age or lifestyle habits, influence its composition [3, 13–16]. In the colon, the phyla Firmicutes and Bacteroidetes compose 90% of the gut microbiota (60–75% and 30–40%, respectively), followed by the phyla Actinobacteria, Proteobacteria and Verrucomicrobia [16–18]. The Firmicutes phylum contains predominantly Gram-positive obligate or facultative anaerobic bacteria and includes, for example, *Lactobacillus* spp., *Clostridium* spp. or *Enterococcus* spp. [5, 16, 17]. The Bacteroidetes phylum contains less genera and predominantly Gram-negative anaerobic bacteria, such as *Bacteroides* spp. or *Prevotella* spp. [5, 16]. The majority of the normal gut microbiota consists of obligate anaerobic bacteria. The latter play a role in inhibiting the growth of other potentially pathogenic bacteria (referred to as pathobionts), mostly composed of aerobic bacteria or facultative anaerobic bacteria such as *Escherichia coli* [19].

### Functions of the gut microbiota

The intestinal microbiota has many functions. First, anaerobic bacteria degrade food polysaccharides, that are fermented into various metabolites including short-chain fatty acids (SCFAs) such as butyrate, acetate and propionate, which are necessary substrates for enterocyte function [20]. It also plays a role in the defence against infections of the digestive tract by a competitive effect between commensal and pathogenic bacteria and in building the local immune defence. In addition, the gut microbiota is closely linked to all our organs and contributes to their normal functioning [21, 22]. This last point, which led to the concept of gut–organ axis, will be detailed below.

### Assessment of the gut microbiota

Gut microbiota can be examined using various methods, the two most commonly used in clinical practice are described below.

16S ribosomal RNA (rRNA) profiling (metataxonomics) [23] provides a taxonomic overview of the bacteria present in a sample and, among others, gives information on microbial richness and diversity [5]. This is a simple, fast and low-cost technique. Limitations include that it gives no information on gene functions and that two organisms with the same 16S rRNA gene sequence could be misclassified [23–25].

A more complete microbial composition can be assessed through unbiased sequencing of all DNA (shotgun metagenomics) present in a sample [23]. This higher resolution approach, although more expensive, allows the identification of bacteria up to species level and provides information on microbial richness, diversity and gene functions [23, 24, 26]. These approaches can be further informed by integrating them with proteins (metaproteomics) and small molecules (metabolomics) profiling.

Finally, these methods produce complex results whose interpretation must be related to a specific research question [27].

### Critical illnesses and the gut microbiota

**Fig. 1:**
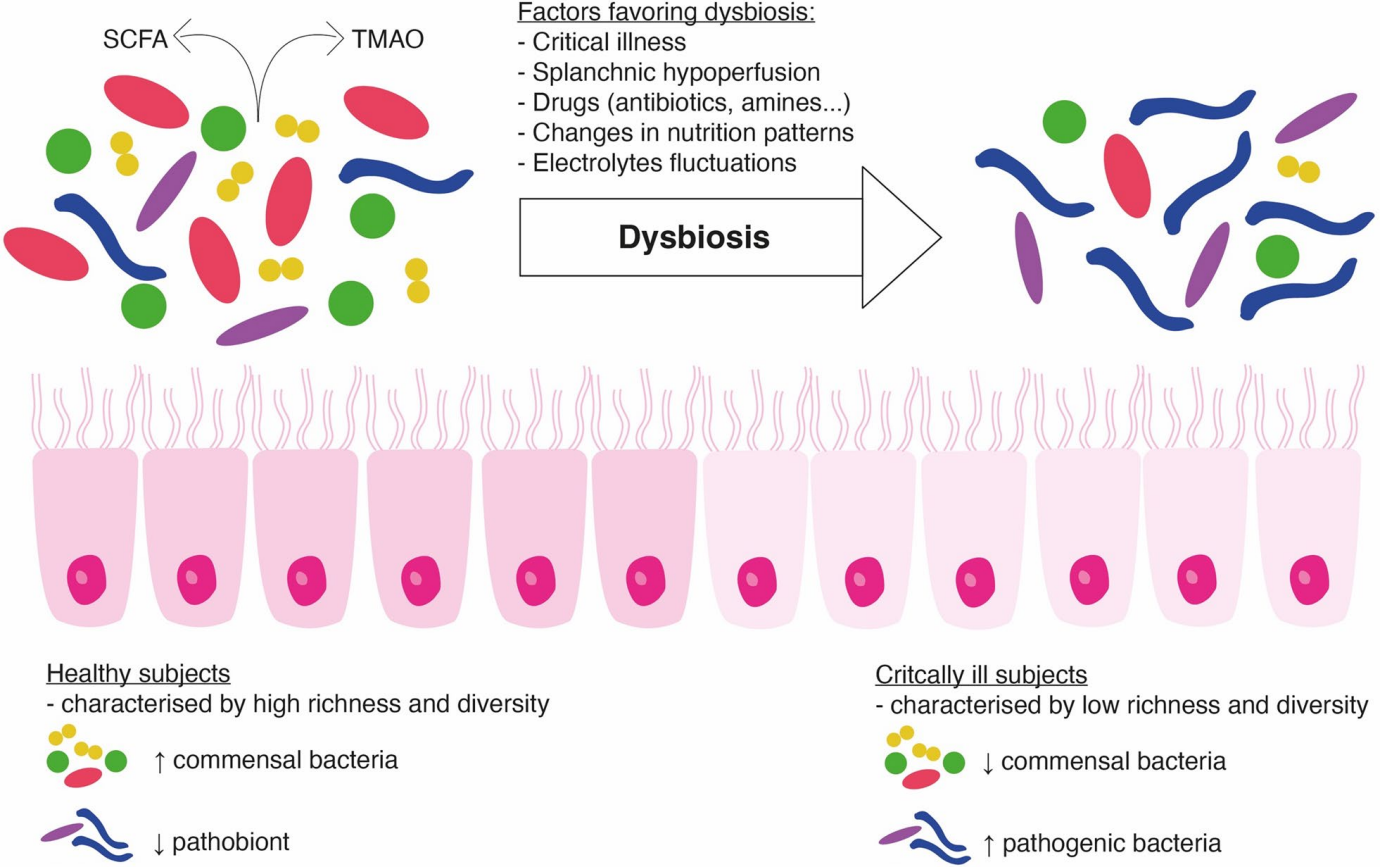
Gut microbiota and dysbiosis in critical illness

Critical diseases are associated with a loss of commensal intestinal bacteria such as Firmicutes or Bacteroidetes and an increase in potentially pathogenic bacteria (pathobionts) such as Proteobacteria [12, 28]. This dysbiosis is determined both by the decrease in diversity and by the change in the ratio of pathogenic bacteria to the detriment of “health-promoting” commensal bacteria (Fig. 1). In some cases, an overgrowth (> 50% relative abundance) of potentially pathogenic genera such as *Enterococcus* spp., *Clostridium difficile, Staphylococcus* spp.*,* can be highlighted [28]. Several indexes exist to identify and define dysbiosis [29]. These changes in microbiota and intestinal homeostasis may occur within the first 48 h following a critical illness and seem to vary according to the patient’s age [12, 30, 31]. A study of 115 critically ill patients comparing the microbiota on ICU admission with that at discharge showed a decrease in Firmicutes and Bacteroidetes phyla, a significant increase in Proteobacteria and an increase in taxa with pathogenic bacteria such as *Enterobacter* spp. and *Staphylococcus* spp. [12]. Another study of mechanically ventilated ICU patients found that the proportion of Bacteroidetes and Firmicutes varies from patient to patient during their stay. This last study also suggested that the Bacteroidetes/Firmicutes ratio could be a predictor of mortality [7]. Intestinal dysbiosis has been shown to be associated with patient susceptibility to nosocomial infections, sepsis, organ failure and even COVID-19 disease severity [32–37].

## Intestinal dysbiosis: how does it work?

### Colonic mucus changes

The intestinal wall is covered with hydrophobic mucus, which is continuously produced by the goblet cells of the mucosa. This mucus protects the enterocytes and colonocytes from digestive enzymes and acts as a barrier against the passage of bacteria and toxins into the bloodstream [28, 32]. In critically ill patients with splanchnic hypoperfusion, mucus production and mucus hydrophobicity decrease, leading to enterocytes injury that promotes cell apoptosis and pathogen translocation [28, 38]. This leads to reduced absorption of nutrients and reduced production of SCFAs and favours diarrhoea [28].

### Intestinal integrity changes and the role of short-chain fatty acids (SCFAs)

The vast majority of knowledge about SCFAs comes from in vitro bench work on human or mice faeces and conclusions from interventional studies with prebiotics [39–42]. The intestinal anaerobic microbiota ferments dietary fibres and produces metabolites such as SCFAs, which help maintain the integrity of the gut barrier and promote the host’s immune response [43]. SCFAs are the primary source of energy for the colonic epithelium and contribute to maintaining functional intercellular junctions. Mostly studied in rodent models, they also play a role in intestinal immunity by controlling the production of T-helper cells, regulatory T cells (Treg), antibodies and cytokines with mainly anti-inflammatory effects [44–46]. SCFAs have also been shown to induce cytoprotective proteins in epithelial cells that help maintain cell viability under stress conditions [32, 47]. Critically ill patients exhibit dysbiosis with a reduction in anaerobic bacteria leading to a decrease in SCFAs concentration, which has been associated with cellular apoptosis, malabsorption, diarrhoea and bacterial translocation [44, 48–50].

### Changes in trimethylamine N-oxide (TMAO) production

TMAO is an important metabolite produced jointly by the intestinal microbiota and the liver [51]. First, trimethylamine (TMA) is produced by the gut microbiota from choline, lecithin and carnitine which are found in food precursors such as meat, fish and eggs [52]. Second, TMA is absorbed and translocated to the liver through portal circulation [51], where TMAO is converted from TMA directly [52]. As the production of TMAO depends on the diversity and composition of the gut microbiota, TMAO levels can change with dysbiosis, resulting often in higher levels [52, 53]. A study in humans showed that broad-spectrum antibiotics suppressed the production of TMAO, which reappeared after the discontinuation of the antibiotics [54], supporting the importance of the gut microbiota in TMAO production. High levels of TMAO have been recognized to be associated with heart failure, atherosclerosis and thrombosis formation [52, 55–59].

### Immune mucosal changes

The gut microbiota plays a crucial role in the development of the immune system and is in constant communication with it [60]. On the one hand, the microbiota promotes the immune system and adapts it to certain conditions; on the other hand, it is tolerated by this adaptive immunity. This occurs through the involvement and recognition of microbe-associated molecular patterns via the toll-like receptor system [61] and through the release of pro-inflammatory cytokines [62], mucus secretion and the formation of SCFAs that activate Treg [20, 63]. This barrier plays an important role in preventing colonization by pathogens and appears to be compromised by antibiotic administration [64].

In order to control its relationship with the microbiota, the immune system limits the contact between the microbiota and epithelial cells, thus limiting the possible translocation of bacteria. This “mucosal firewall” consists of epithelial cells, IgA secretion, antimicrobial peptides and immune cells [65, 66]. Alteration of the microbiota can lead to dysregulation of the immune system, including a decrease in IgA and T cell levels, favouring bacterial infection [5, 67].

## Intestinal dysbiosis in critically ill patients: pathophysiological concepts

Multiple environmental changes take place during critical illness, during which there is selective pressure due to splanchnic hypoperfusion in the context of shock, inflammation, impaired immunity, change in diet, medications and decreased intestinal motility [27, 33, 68]. All these conditions could contribute to the development of intestinal dysbiosis.

### Factors favouring dysbiosis in an intensive care setting

Several factors influence the change in microbiota and its virulence. First, during critical illness, transit time is prolonged, leading to a reduction in bacterial excretion, which is known to be associated with bacterial overgrowth [6, 69]. The slowing down of intestinal transit time may be due to electrolyte fluctuations and the frequent use of sedatives and opiates in the ICU [6].

Second, many drugs commonly administered in the ICU can affect the composition of the gut microbiota, such as antibiotics but also non-steroidal anti-inflammatory drugs, beta-blockers, amines, or proton pump inhibitors [70–73]. A possible explanation for this last drug family is that the gut pH exerts selection pressures on bacteria, which cannot all grow in the same acidic environment [74, 75]. The dysbiosis induced by proton pump inhibitor has been associated with an increased risk of *Clostridium difficile* infection [71, 76, 77].

The effects of antibiotics on microbiota depend on many factors, including the class of antibiotic therapy and its route of elimination. In general, antibiotics alter the commensal flora and its diversity and could select and/or promote the growth of resistant microorganisms [5, 78].

Finally, another important factor is the change in nutrition patterns. Critically ill patients are often starving and are fed with enteral nutrition (EN) or parenteral nutrition (PN). Little is known about the effects of EN and PN on the human gut microbiota. However, a study in children in ICU confirmed the findings of murine models, that exclusive PN was associated with significant dysbiosis [79, 80]. In contrast, an in vitro study on human faecal samples has shown that EN promotes the growth of commensal microbiota, with intraindividual differences depending on the enteral formula [81]. Nutritional therapy seems to have significant impacts on the gut microbiota. NE appears to be a protective factor for the gut microbiota, whereas periods of starvation or total PN should be avoided as they may affect the integrity of the gut microbiota [28, 82–84].

### When the normal gut flora becomes pathogenic

It is assumed that bacteria are able to sense their environment including the density and diversity of other bacteria [32]. In fact, depending on the intestinal lumen environment, intestinal bacteria either continue colonizing or become pathogenic. Many bacteria express virulence genes through a system called quorum sensing [32]. This system causes the bacteria's virulence genes to be expressed only when a certain bacterial density is reached that can overwhelm the host, and only when a negative environmental change is perceived, such as nutrient deficiency or specific treatment with opiates [6, 32, 85]. Indeed, a study showed that in patients with long ICU stays, “normal” microbiota was replaced by ultra-low-diversity communities of resistant pathogens whose virulence varied depending on the local environment, such as exposure to opiates [85]. Another study has shown that during acute stress associated with intestinal ischaemia/reperfusion, the production of dynorphin, a natural human opioid, was increased. In this study, exposure of *Pseudomonas aeruginosa* to dynorphin activated the quorum sensing system, which enables bacteria to recognize stress in the host, become pathogenic and take advantage of the host weaknesses [86].

Furthermore, the electrolytes levels also seem to influence gut microbiota. For example, local phosphate levels have been suggested to influence gut microbiota virulence [87, 88]. In this context, a study on mice models has shown that *Pseudomonas aeruginosa* and other pathogens can develop a lethal phenotype in the case of hypophosphatemia [87–89].

The main factors that influence the microbiota in critical illness are as follows: the critical illness itself, the host status, the drugs and the nutrition administered [27].

### Sepsis and microbiota

Numerous ICU patients have severe infections. Although the specific mechanisms are not yet fully identified, the gut microbiota appears to play a role in the pathophysiology of sepsis [90, 91]. This is partly due to the fact that critically ill patients often receive a wide range of medications, which affect gut microbiota diversity [90], and partly because of the patients’ precarious condition, which can lead to hypoxic lesions, inflammation, disruption of epithelial integrity, dysmotility, changes in intraluminal pH or impaired immune function in the gut [92]. There are some characteristic patterns of gut microbiota associated with sepsis. In a multicentre study, the microbiota of ICU patients with sepsis showed an increased abundance of microbes closely associated with inflammation, such as *Parabacteroides, Fusobacterium* and *Bilophila* species [93]. Other studies showed that the gut loses important bacterial genera, including *Faecalibacterium* spp., *Prevotella* spp., *Blautia* spp. and *Ruminococcaceae* spp. [7, 85, 94], which are known to produce SCFAs [20]. Furthermore, it has been shown that certain antibiotic-resistant species prevalent in sepsis, such as *Enterococcus* spp. or *Clostridia* spp., are associated with unfavourable outcomes [85, 93, 95]. The gut microbiota is thought to influence sepsis not only through bacterial translocation [14, 96] and through the prevention of colonization by multi-resistant pathogens [64], but also by regulating the immune system [97, 98]. Laboratory data show greater bacterial spread, higher levels of inflammation and organ failure, and higher mortality in germ-free mice during sepsis compared to healthy mice, likely due to a less pronounced immunomodulatory response [97].

### Modulation of the gut microbiota

Prebiotics, probiotics, synbiotics and faecal microbiota transplantation (FMT) are the most studied specific treatments for modulating gut microbiota.

Prebiotics are defined as undigested food substrates, such as fibres, inulin or oligosaccharides, that are used by the commensal gut microbiota after ingestion and provide health benefits [99]. A few studies on prebiotics showed that administration of fibre in ICU patients could improve dysbiosis, increase SCFAs production and reduce hospital length of stay [100, 101], while other studies showed contrasting results [102, 103].

Probiotics are living microorganisms that help maintain the balance of gut microbiota and improve the health of the host. Synbiotics is the concomitant administration of prebiotics and probiotics [99]. Previous studies have shown a possible effect of probiotics in reducing the incidence of ventilator-associated pneumonia (VAP) [104, 105]. However, subsequent randomized controlled trials (RCTs) yielded conflicting results [106, 107]. The results of these studies cannot be generalized because the probiotics used and their dosage varied from study to study, which is a recurrent problem in studies comparing probiotics. Other studies using other genera, species, strains or doses are expected to clarify this issue [107]. Although the use of probiotics is an attractive microbiota-targeted therapy, they are not without risk, particularly in ICU patients, where Lactobacillus bacteraemia has been described following probiotic administration [108].

Recently, there has been increasing interest on FMT, which consists of transplanting an autologous or donor stool through colonoscopy, oral capsules or enteral feeding tube to restore a healthy microbiota. FMT has for example been proposed as an alternative treatment for severe or recurrent *Clostridium difficile* colitis [109, 110]. In the ICU, there are case reports in septic patients with multiple organ failures and suspected dysbiosis highlighting successful FMT in these patients [111, 112]. The physiopathological hypotheses are that FMT increases SCFA-producing bacteria, which could help restore the systemic immune response and allow the clearance of the sepsis pathogen [113]. However, FMT is not without risk in ICU patients and is still an experimental treatment.

As knowledge about gut microbiota keeps growing at an impressive rate, we can anticipate further definitions of the value and use of specific treatments modulating gut microbiota.

## Interaction of the gut microbiota with key organs: the concept of the gut–organ axis

**Fig. 2:**
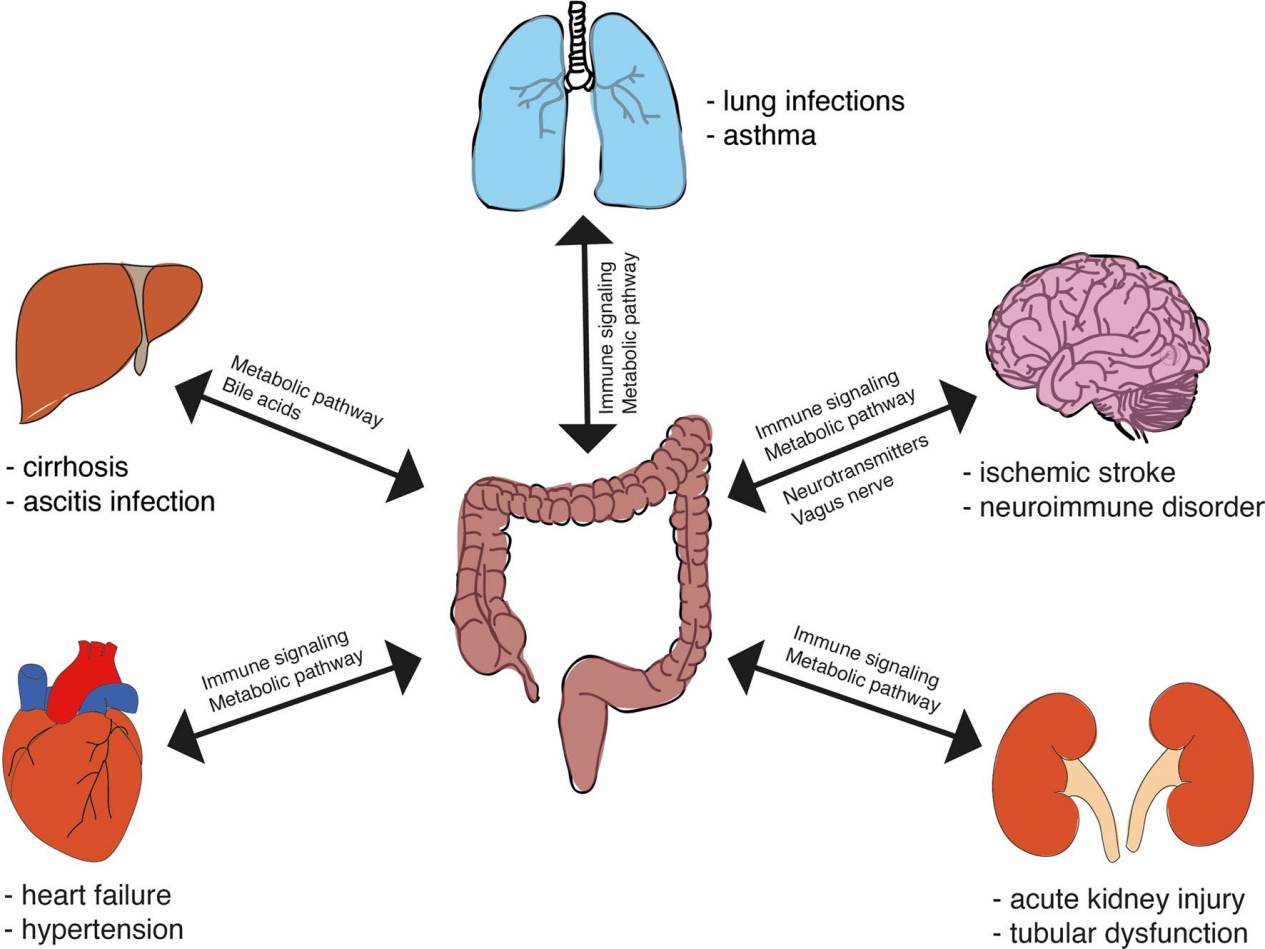
Gut–organ axis

As gut microbiota interacts with other organs, the concept of gut–organ axis is explored in this section. Figure 2 illustrates the different gut–organ axes and provides examples of diseases associated with an alteration of the gut microbiota.

### Gut–brain axis

Gut–brain axis is an important, constant bidirectional communication system [114–116], taking place via immunological, endocrine, neural and metabolic pathways [117].

Immune signalling is mediated by cytokines (IL-1, IL-6), that are produced in the gut, travel through the bloodstream and cross the blood–brain barrier [118, 119]. These cytokines then influence one of the most powerful activators of the stress system, the hypothalamic–pituitary–adrenal axis [118, 119].

The gut microbiota has been shown to interact with the brain via neurotransmitters and the vagus nerve. The neurotransmitters produced and consumed by the gut include dopamine, norepinephrine, GABA and serotonin [120]. Some bacteria have been shown to express more neurotransmitters, such as *Lactobacillus rhamnosus*, which is associated with neurological GABA secretion. Interestingly, the vagus nerve appears to recognize metabolites of the gut microbiota and responds through a cholinergic pathway that appears to reduce intestinal inflammation and intestinal permeability, thus modulating the gut microbiota [120–122]. Recent studies also suggested that alterations of these neurotransmitters by the microbiota have an impact on the onset and development of neurological diseases such as ischaemic stroke or neuroimmune diseases [123]. The vagus nerve also seems to be activated by SCFAs [124–126].

Metabolic components also serve as communication pathways between the brain and the gut microbiota. For example, it has been shown that colonization with *Bifidobacterium infantis* leads to higher plasma tryptophan levels and secondarily to higher central serotonin levels [127, 128].

The gut–brain interaction has been demonstrated in neurocritically ill patients. Indeed, their gut microbiota appears different from that of healthy subjects and dysbiosis increases with ICU length of stay [129]. Furthermore, an increased abundance of *Enterobacteriales* and *Enterobacteriaceae* in the first week after ICU admission was associated with 180-day mortality in these patients [129]. Another well-studied clinical example is acute ischaemic stroke, which leads to intestinal ischaemia and dysbiosis, which in turn exacerbate cerebral infarction by enhancing systemic inflammation [130, 131]. In addition, dysbiosis is associated with poor outcomes following acute ischaemic stroke as it interacts with the brain through all of the above mechanisms [56, 132, 133].

Different stroke dysbiosis indexes are being explored to characterize gut microbiota in these patients and correlate them with patient outcomes [134].

### Gut–lung axis

The gut microbiota constantly interacts with the lung microbiota [135]. In fact, the lung microbiota has been shown to change when a newborn's diet is altered [136].

Since the microbiota is known to have an effect on local immunity, it is thought to play a role in pulmonary immunity as well. The immune response of the lungs can probably be modulated in the following way. Besides portal circulation, drainage of the gastrointestinal tract also occurs via lymph nodes, which drain into the thoracic duct and then into the subclavian vein. The first capillary bed that then filters the chyle is the pulmonary capillary bed [6].

SCFAs seem to play a role in immunity by reducing lung inflammation through induction of Treg [137]. Furthermore, dysbiosis with an increased ratio of Firmicutes/Bacteroidetes species is also associated with increased IL-17 and IL-22 responses in the lung, which could lead to airway hyperreactivity [137].

In mice models, the gut microbiota appears to have a protective effect in severe lung infections, as several studies showed that germ-free mice had increased mortality after lung infection with *Klebsiella pneumoniae, Streptococcus pneumoniae or Pseudomonas aeruginosa* [138, 139]*.* One of the possible mechanisms is that the phagocytic capacity of macrophages decreases in germ-free mice [97].

The importance of the gut–lung axis and its therapeutic potential is also supported by a few interventional studies in humans. For example, some studies have shown that the use of probiotics could reduce the risk of VAP in the ICU [48, 140].

### Gut–heart axis

Gut microbiota and the cardiovascular (CV) system also interact bidirectionally [141].

Dysbiosis has recently been associated with CV risk factors and diseases such as atherosclerosis, obesity, diabetes, hypertension or coronary artery disease [142]. On the one hand, CV diseases lead to dysbiosis, while on the other hand, the gut microbiota affects the CV system through various metabolites, including TMAO and SCFAs [142].

High TMAO levels have been shown to be associated with CV disease [143, 144] and with an increased risk of serious CV events (e.g. death, myocardial infarction and stroke) and heart failure [54, 145–147]. The gut microbiota has also been shown to influence platelet hyperresponsiveness and blood clot formation through the production of TMAO [56].

SCFAs may play a role in blood pressure regulation by influencing renin secretion via the G-protein-coupled receptor pathway [148], and different studies suggest a link between gut microbiota and hypertension [148, 149]. Moreover, dysbiosis is associated with lower butyrate production, leading to increased intestinal permeability and systemic inflammation, promoting atherosclerosis and heart failure [141].

Patients with heart failure also experience relative splanchnic hypoperfusion, leading to oedema of the intestinal wall and impaired function and permeability of the intestinal epithelium, which could lead to dysbiosis [150, 151]. This dysbiosis is thought to be associated with increased inflammation, which can exacerbate acute heart failure [150].

In CV surgery patients, a small longitudinal study has shown marked changes in gut microbiota in patients admitted to the ICU, with more complications in patients with the most pronounced dysbiosis [30].

In summary, an imbalance of the gut microbiota metabolites seems to contribute to the development or exacerbation of CV diseases. This has led to new research and clinical opportunities, with a focus on the use of TMAO as a potential biomarker.

### Gut–kidney axis

So far, several mechanisms have been identified (e.g. SCFAs, TMAO) that could explain how the gut microbiota interacts with the kidney, but knowledge in humans and in critical care situations remains scarce [152]. First, regarding the SCFAs mechanism, Andrade-Oliveira et al. [153] showed that mice treated with acetate-producing bacteria had better outcomes after acute kidney injury (AKI) by regulating inflammation. Second, high levels of TMAO have been recognized as a risk factor for chronic kidney disease (CKD). In murine models, dysbiosis can lead to an increase in circulating TMAO, which in turn can cause kidney interstitial fibrosis [154]. TMAO levels have also been shown to be higher in patients with CKD compared to healthy subjects and associated with poor prognosis [155].

Intestinal bacteria are known to affect dendritic cell activity on intestinal T cells as well as on peripheral Treg differentiation. It has been shown that the amount of CD4 T-helper cells producing pro-inflammatory IL-17 is higher in patients with autoimmune kidney disease [156].

Increased inflammation also affects kidney function. In sepsis and subsequent dysbiosis, there is an increased intestinal permeability and silent translocation of bacteria and toxins into the bloodstream. This increases inflammation and promotes the switch to renal aerobic glycolysis, leading to a decrease in ATP stores and ultimately to mitochondrial and cellular damage in the kidney [157].

Finally, urea works both ways. On the one hand, it accumulates in AKI and promotes intestinal damage [157]. On the other hand, dysbiosis produces more uremic toxins that can lead to tubular dysfunction [156].

### Gut–liver axis

Bidirectional interactions between gut microbiota and the liver occur through continuous exchange via the portal circulation as well as the biliary enterohepatic cycle [158].

Via the portal circulation, the liver is directly exposed to molecules absorbed through the intestinal mucosa. A study [159] indicated an inverse correlation between SCFA levels and the severity of portal hypertension, the degree of endotoxemia and systemic inflammation, emphasizing the role of gut microbiota in gut–liver interactions and in the progression of liver pathologies such as cirrhosis [160]. The gut–liver interaction was also confirmed in another study [161] which demonstrated a negative correlation between the abundance of endogenous bacteria and inflammatory markers in patients with alcohol use disorders.

Moreover, non-alcoholic fatty liver disease and its severity have also been associated with TMAO levels [53, 59]. TMAO may affect triglycerides levels in the liver and influence their metabolism [162].

The biliary enterohepatic cycle is another central protagonist allowing the liver to communicate with the gut by releasing bile acids (BAs) and other bioactive mediators through the biliary tract. Furthermore, almost 5% of the BAs are metabolized into secondary BAs which exert direct control on microbiota by inhibiting microbial overgrowth [163]. Indeed, dysbiosis is thought to lead to an imbalance between primary and secondary BAs which causes an additional metabolic burden on the liver.

The role of the microbiota has led to the development of a *cirrhosis–dysbiosis ratio* (CDR) to classify the severity of dysbiosis in cirrhotic patients, as reported by Bajaj et al. [160, 164]. The latter pointed out that low CDR (i.e. more severe dysbiosis) was associated with decompensated cirrhosis, organ failure and death [160].

Gut–liver interplays are of interest in the ICU, especially in the context of liver transplantation and hepatic encephalopathy (HE). Liver transplantation seems to improve dysbiosis in cirrhotic patients and establish better cognitive status [165]. Moreover, a phase I RCT highlighted that FMT in cirrhotic patients with HE could improve dysbiosis and cognitive state [166].

## Conclusion

The gut microbiota is in constant communication with key organs of our organism and strongly influences them. According to the latest evidence, gut microbiota could be considered as an organ and its failure, manifested by dysbiosis, as an organ failure, which is possibly associated with poor clinical outcomes. The exact roles and contributions of the gut microbiota and its interactions with the various organs are an intense and challenging area of research, and much remains to be discovered. Another aspect that should not be neglected is that the composition of the gut microbiota is influenced by genetic and non-genetic factors such as lifestyle, diet, but also by diseases and their treatments. Further research on the gut microbiota is needed to better understand these processes, and to offer new opportunities for disease prevention, management and therapy, especially in critical care where multi-organ failure is often the focus.

## Data Availability

Not applicable.

## Abbreviations

SCFA: Short-chain fatty acid
ICU: Intensive care unit
TMAO: Trimethylamine N-oxide
TMA: Trimethylamine
EN: Enteral nutrition
PN: Parenteral nutrition
CV: Cardiovascular
RCT: Randomized controlled trial
VAP: Ventilator-associated pneumonia
FMT: Faecal microbiota transplant
CKD: Chronic kidney disease
BA: Bile acid
CDR: Cirrhosis–dysbiosis ratio
